# Vortex-assisted low density solvent and surfactant based dispersive liquid–liquid microextraction for sensitive spectrophotometric determination of cobalt

**DOI:** 10.1039/c7ra11896a

**Published:** 2018-02-14

**Authors:** Patiwat Chaiyamate, Ketsarin Seebunrueng, Supalax Srijaranai

**Affiliations:** Materials Chemistry Research Center, Department of Chemistry, Center of Excellence for Innovation in Chemistry, Faculty of Science, Khon Kaen University Khon Kaen 40002 Thailand supalax@kku.ac.th +66 43 009700 ext. 42175

## Abstract

This study describes the development of vortex-assisted low density solvent and surfactant based dispersive liquid–liquid microextraction (VALS-DLLME) for Co(ii) prior to its spectrophotometric detection. The method consisted of the complexation of Co(ii) with pyrocatechol violet (PV) followed by the preconcentration of the Co(II)–PV complex using VALS-DLLME and then an absorption measurement at 600 nm. The optimum conditions for complex formation were a 1 : 3 mole ratio of Co(ii) and PV at pH 7.5, while the conditions for VALS-DLLME were 300 μL 1-dodecanol as extraction solvent, and 300 μL acetonitrile as dispersive solvent under a vortex for 20 s with the addition of cationic surfactant (0.02 mmol L^−1^ CTAB). Under the optimum conditions, good linearity was in the range of 0.1–10 mg L^−1^, the enrichment factor (EF) was 13.5 and the low limit of detection (LOD) was 0.04 mg L^−1^. The method was applied to the analysis of Co(ii) in water, green leaf vegetable and vitamin B_12_ samples. The proposed method provided good recoveries in the range of 86–104%, which were comparable to those obtained from flame atomic absorption spectrophotometry.

## Abstract 2

The formation of a Co(II)–PV complex and the VALS-DLLME procedure.

## Graphical abstract

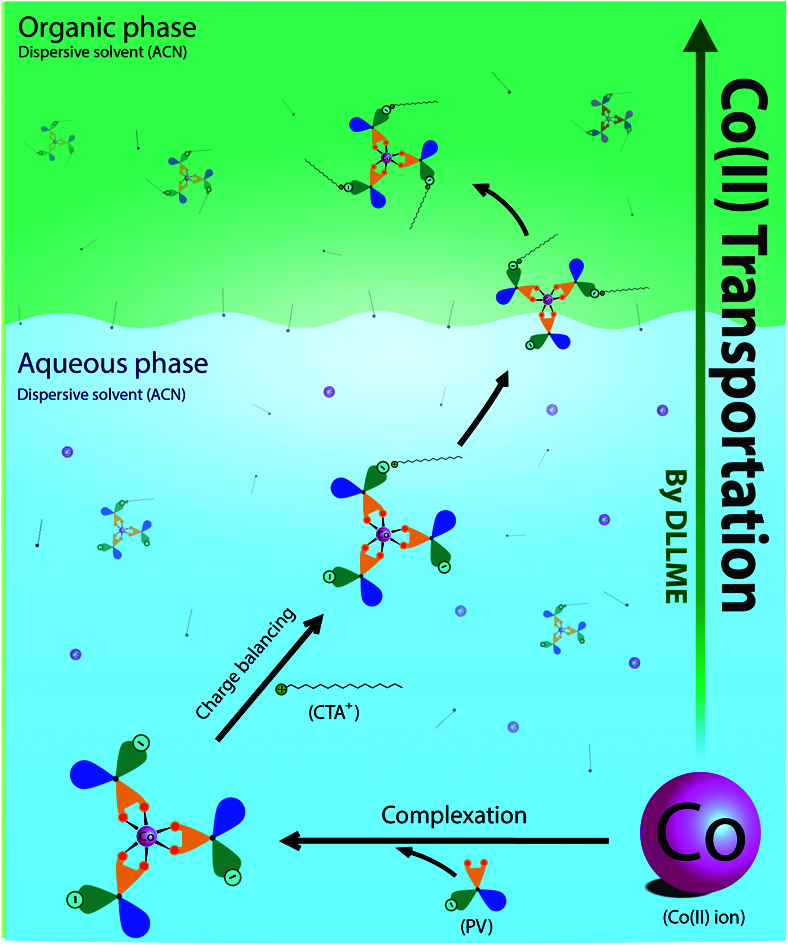

## Introduction

Cobalt is an essential element in the human body since it plays an important role in many vital processes including blood formation, synthesis of hormones, hemoglobin, neurotransmitters and other compounds, such as bile acids and DNA.^1^ Moreover, cobalt influences the functionality of vitamins such as vitamin C (ascorbic acid) and vitamin B_12_.^2^ Generally, cobalt is supplied to humans through food and drink. The determination or monitoring of cobalt in body fluids is important for controlling nutritional deficiencies and also the prevention of toxicity from the exposure to a high amount of cobalt. Deficiency of cobalt leads to several diseases such as pernicious anemia. On the other hand, toxicities to humans from the intake of a large amount of cobalt are vasodilation, flushing and cardiomyopathy.^3,4^ Thus, the development of analytical methods for trace cobalt determination is still required.

The sensitive and selective method for the determination of metal ions including cobalt is atomic spectrometry; both atomic absorption spectrometry (AAS) and inductive couple plasma optical emission spectrometry (ICP-OES) have been widely used. However, these techniques are sophisticated and expensive. An alternative technique for detection of metal ions is molecular spectrometry. Visible spectrometry has been accepted as a simple and cost effective instrumental method for chromophore analytes. The unique property of metal complexes enables the determination of metal ions by visible spectrometry. Pre-derivatization of the metal ions by complexation is, therefore, necessitated before their spectrometric determination. Complexation provides not only selectivity from appropriate ligands for metal ions but also sensitivity from high molar absorptivity of the obtained complexes. There are a number of ligands used for the determination of cobalt by spectrometry such as ninhydrin,^5^ hydroxytriazene^6^ and 5-(2-benzothiazolylazo)-8-hydroxyquinolene.^2^ However, the use of visible spectrometry is still limited by its low sensitivity, especially for trace analysis. To increase the detection sensitivity, preconcentration is recommended.

Dispersive liquid–liquid microextraction (DLLME) has become the most popular technique in liquid phase microextraction since its introduction in 2006 by Assadi and co-workers^7^ due to many advantages such as simplicity, high enrichment factors and rapidness. DLLME is based on ternary solvent system including an aqueous solution, a water immiscible solvent (extraction solvent) and a water miscible solvent (dispersive solvent).^8,9^ A mixture of an extraction solvent and a dispersive solvent is injected rapidly into an aqueous solution. Then, a stable emulsion is formed containing fine droplets of the extraction solvent dispersed in the aqueous solution resulting in a large increase in contact area between the two phases. The analytes are easily transferred into the extraction phase, thus enriched. After extraction, the emulsion is separated into two phases using centrifugation and the extraction phase is subjected for analysis. DLLME has been successfully applied for various analytes including organic compounds such as pesticides^10–12^ biogenic amine^13^ and metal ions.^14,15^ However, there are some limitations in DLLME including (i) the use of hazardous chlorinated solvents having density higher than water, (ii) the use of large amounts of dispersive solvents, resulting in a decrease in partition coefficients of analytes and (iii) requirement of extra time for the centrifugation step.^16–18^ To overcome these drawbacks and to achieve green extraction, various methods based on DLLME have been proposed such as using other extraction solvent groups that are more environmentally friendly than conventional DLLME,^19–21^ using external forces (ultrasound and vortex) in the dispersion process^22–24^ and omitting the centrifugation step to reduce time consumption.^16,25,26^

To our knowledge, the reports on DLLME as a preconcentration technique for the spectrometric analysis of cobalt are mostly based on the analysis by flame atomic absorption spectrometry (FAAS).^27–30^ Only three articles have been reported on the use of DLLME coupled with ultraviolet-visible (UV-vis) spectrophotometric detection of cobalt.^26–28^ For instance, 1-(2-pyridylazo)-2-napthol (PAN)^31,32^ and newly synthesized cinnamoyl pyrones^33^ were used as ligands for the complexation of cobalt prior to DLLME and UV-vis detection.

This research was aimed at the development of a selective and sensitive spectrometric method for the determination of cobalt. Cobalt was first derivatized *via* complexation with pyrocatechol violet (PV). After that, the cobalt complex was enriched by the modified DLLME, named vortex-assisted low density solvent and surfactant based DLLME (VALS-DLLME).

## Experimental

### Chemicals and reagents

All chemicals used are of at least analytical reagent grade. Cobalt chloride hexahydrate (CoCl_2_·6H_2_O), metal ions studied for interferences, pyrocatechol violet and nitric acid were obtained from Carlo-Erba, France. 1-Undecanol, 1-dodecanol, dodecyltrimethyl ammonium bromide (DTAB), trimethyltetradecyl ammonium bromide (TTAB), and cethyltrimethyl ammonium bromide (CTAB) were purchased from Sigma-Aldrich (USA). 1-Octanol was obtained from Panreac (Spain). Methanol (HPLC grade) and acetonitrile (HPLC grade) were obtained from Merck (Germany). Acetone was purchased from Q-rac (Malaysia). Sodium sulphate anhydrous was purchased from Fluka (Japan). Sodium chloride, sodium hydrogen phosphate and sodium dihydrogen phosphate were purchased from Ajax Finechem (Australia). Aluminium chloride was obtained from Ajax Finechem Pty Ltd (New Zealand). All solutions were prepared using deionized water with the resistivity of 18.2 MΩ cm from RiOs™ Type I Simplicity 185 (Millipore Waters, USA). Stock solution of standard Co(ii) 100 mg L^−1^ (or 1.695 mmol L^−1^) was prepared and used throughout.

### Instruments

Absorbance measurements and spectra recording were performed on a spectrophotometer (Agilent Technologies Cary 60 UV-Visible Spectroscopy System, Germany). A 1 cm micro quartz cell was used throughout the experiments. An analytical balance (Scaltech, Germany) was used. A pH meter (Model 251, Denver Instrument, USA) was used for pH measurement. Vortex (Scientific Industries, INC., USA) was used for mixing.

Quantitative determination of Co(ii) was also performed on a flame atomic absorption spectrophotometer (Perkin Elmer Instrument AA Analyst 100, England).

### Sample preparation

#### Water samples

Water samples including tap water, groundwater, surface water, agricultural water and waste water were filtered through Whatman (no. 42) filter paper before analysis.

#### Green leaf vegetable samples

Chinese cabbage, mint, spinach, cabbage and kale were investigated. The samples were cleaned with tap water and deionized water, respectively. After that, they were dried at room temperature and weighed. Then, they were burned to ash in a muffle furnace at 450 °C. Accurately, 0.1 g of ash samples was dissolved in 6 mol L^−1^ HCl, diluted to 50 mL in a volumetric flask and then were filtered through Whatman (no. 42) filter paper before analysis.^2,34^

#### Vitamin B_12_ samples

Five different brands of vitamin B_12_ samples were purchased from pharmaceutical drug stores in Khon Kaen, Thailand. Five tablets of each sample were weighed and ground by a mortar and a pestle. The average accurate weight of samples was digested by 8 mL of 1 : 1 HNO_3_, heated to near dryness and subsequently digested by 8 mL of 1 : 1 HCl. The residue was dissolved by adding an appropriate amount of water to 50 mL and filtered through Whatman (no. 42) filter paper before analysis.

### Preconcentration procedure and determination

Standard solution of Co(ii) or the sample solution 5 mL was placed in a 10 mL volumetric flask. Aliquot 2.30 mL of 0.26 mmol L^−1^ PV, 50 μL of 1 mol L^−1^ phosphate buffer (pH 7.5), 200 μL of 1 mmol L^−1^ CTAB and 0.2 g Na_2_SO_4_ were added. Then, a mixture of 300 μL of 1-dodecanol (extraction solvent) and 300 μL of acetonitrile (dispersive solvent) was rapidly injected into the solution. After that water was added to make a final volume of 10 mL. The solution was then mixed using a vortex mixer at 3200 rpm for 40 s. The Co(II)–PV complex was extracted into the fine droplet of the extraction solvent. After leaving the solution to stand, the extraction phase at the top of the solution was collected and diluted with 600 μL of methanol and detected by a spectrophotometer at 600 nm.

## Results and discussion

### Optimization for the formation of Co(II)–PV complex

#### Absorption spectra

**Fig. 1:**
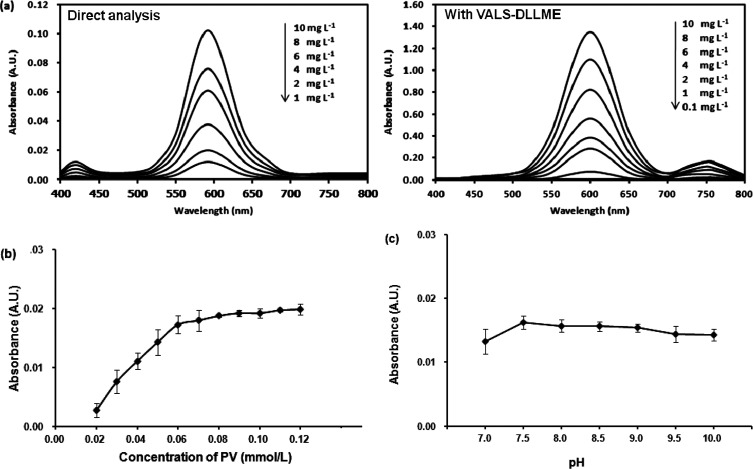
(a) Absorption spectra of Co(ii)–PV complex at different concentrations of Co(ii) obtained from direct analysis (left) and with VALS-DLLME (right). Conditions of complexation: molar ratio (Co(ii) : PV) of 1 : 3, phosphate buffer pH 7.5. Condition of VALS-DLLME: 300 μL 1-dodecanol, 0.02 mmol L^−1^ CTAB, 300 μL acetonitrile, 0.2 g Na_2_SO_4_, vortex at 3200 rpm for 40 s. (b) Effect of the PV concentration. Conditions: 1.00 mg L^−1^ Co(ii) and phosphate buffer pH 7.5. (c) Effect of pH. Conditions: 1.00 mg L^−1^ Co(ii) and 0.06 mmol L^−1^ PV in phosphate buffer.

The Co(II)–PV complex has blue color with the maximum absorption wavelength at 590 nm, while PV is yellow color with the maximum absorption wavelength at 445 nm. The absorption spectra of the Co(II)–PV complex at different concentrations of Co(ii) are shown in (Fig. 1(a); direct analysis).

#### Effect of PV concentration

The influence of PV concentration was studied using 1.00 mg L^−1^ Co(ii) (or 0.0169 mmol L^−1^ Co(ii)) by varying PV concentrations from 0.02 to 0.12 mmol L^−1^. The results (Fig. 1(b)) reveal that the maximum absorbance of Co(ii)–PV complex was achieved at the 0.06 mmol L^−1^ PV. Therefore, the mole ratio is 0.0169 mmol L^−1^ Co(ii) to 0.06 mmol L^−1^ PV providing 1 : 3.5 implying 1 : 3 mole ratio of M : L, which is in good agreement with an earlier report.^35^

#### pH study

The effect of pH on the Co(ii)–PV complex formation was investigated in the range of 7.0–10.0. In the studied pH range, PV exists in H_2_L_2_; whereas, the blue color metal complex of PV can be either ML_2_ or MHL.^36,37^ As shown in Fig. 1(c), the absorbance increased from pH 7.0 to 7.5. At higher basicity, the pH did not affect the absorbance. Such effect of pH was also reported previously.^37^ Therefore, pH 7.5 was used throughout this study for the complex formation of Co(ii) and PV which phosphate buffer was employed.

Moreover, the stability of the Co(II)–PV complex was studied by leaving the complex solution for 1 hour before absorption measurement every 10 min. The results indicated that the Co(ii)–PV complex was stable within 1 hour of the studied time.

### Optimization of the VALS-DLLME

The preliminary study for extraction of Co(ii)–PV complex by DLLME was unsuccessful as the obtained Co(ii)–PV complex is anionic. Thus, it is necessary to neutralize the negative charge of Co(ii)–PV complex by an addition of cationic surfactants into the solution which led to the successful extraction of the complex from the solution. However, after VALS-DLLME, the greenish blue solution of the complex was obtained, showing that there was a bathochromic shift of the maximum absorption wavelength from 590 nm to 600 nm (Fig. 1(a); with VALS-DLLME). In addition, the dominant bathochromic shift was observed for PV,^36^ by which the maximum absorption wavelength shifted from 445 nm to 720 nm. To obtain the highest extraction efficiency, parameters affecting the efficiency of VALS-DLLME were studied and optimized using 1.00 mg L^−1^ Co(ii) and 0.059 mmol L^−1^ PV throughout the subsequent studies.

#### Effect of type and concentration of surfactant

**Fig. 2:**
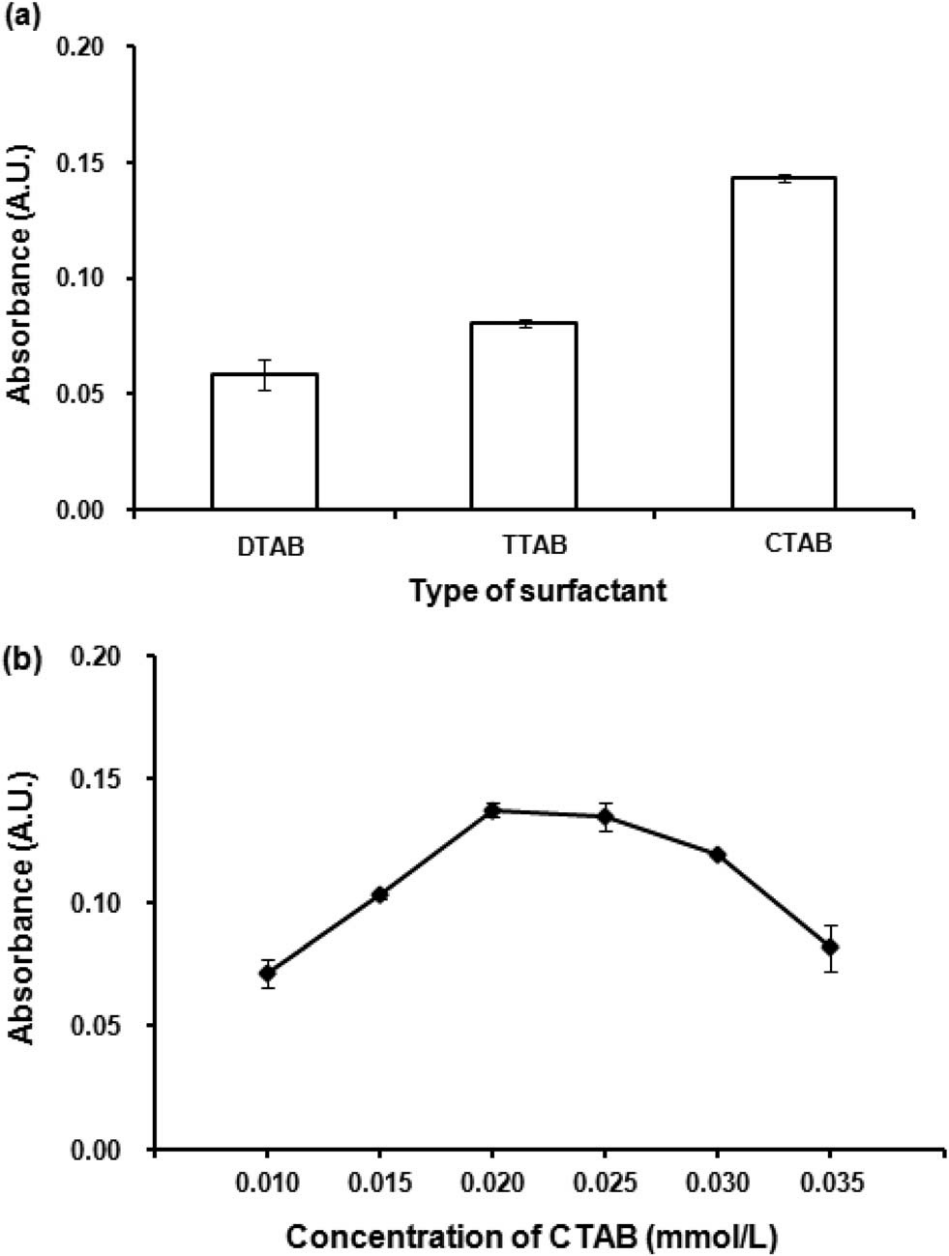
(a) Effect of surfactant. Conditions for VALS-DLLME: 300 μL 1-dodecanol, 0.02 mmol L^−1^ surfactant, vortex at 3200 rpm for 40 s. (b) Effect of CTAB concentration. Conditions: as described in (a) except CTAB concentrations were varied.

As the Co(ii)–PV complex is negatively charged, to accomplish the extraction, the charge balancing is required. In this study, cationic surfactant was employed as a charge balancing agent. The cationic surfactant not only neutralize the negatively charge of the complex by its polar head (positive charge) but also solubilize the complex from its non polar tail. Three cationic surfactants with different carbon chains including DTAB (C = 12), TTAB (C = 14) and CTAB (C = 16) were investigated. Fig. 2(a) shows the effect of the cationic surfactants on the extraction efficiency. It is clearly seen that CTAB gave the highest absorbance. This may be due to the highest hydrophobicity from the longest chain of CTAB. Consequently, CTAB was chosen for further studies.

Different concentrations of CTAB in the range of 0.010 to 0.035 mmol L^−1^ were investigated. Fig. 2(b) shows the dependence of absorbance upon the concentrations of CTAB. The absorbance increased with an increasing concentration of CTAB from 0.010 mmol L^−1^ to 0.020 mmol L^−1^. This can be explained by insufficient balancing of the anionic complex with low concentration of the positively charged CTAB. In addition, it was observed that the aqueous solution was still of the greenish blue color when the concentration of CTAB was less than 0.020 mmol L^−1^. The highest absorbance was obtained at 0.020 mmol L^−1^ CTAB and then slightly decreased. This is probably attributable to an excess of surfactant, thus increasing the solubility of the complex in an aqueous phase.^38,39^ The concentration of CTAB at 0.020 mmol L^−1^, which provided the highest absorbance was therefore selected for further experiments.

#### Effect of type and volume of the extraction solvent

**Fig. 3:**
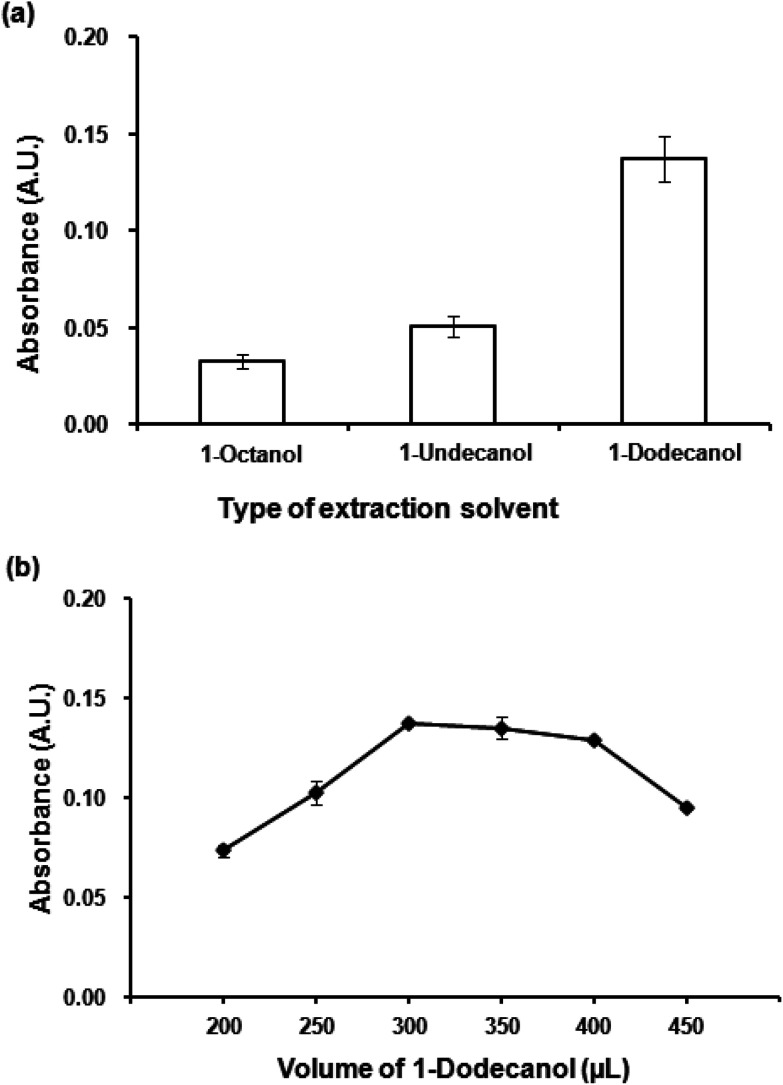
(a) Effect of extraction solvent. Conditions for VALS-DLLME: 300 μL 1-dodecanol, 0.02 mmol L^−1^ CTAB, vortex at 3200 rpm for 40 s. (b) Effect of extraction solvent volume. Conditions: as described in (a) except volumes of 1-dodecanol were varied.

The choice of extraction solvent is an important parameter to obtain an efficient extraction procedure. In conventional DLLME, generally hazardous solvents having density higher than water are used for the extraction. To fulfill the green extraction concept, three low density organic solvents including 1-octanol (log *K*_ow_ = 3.15), 1-undecanol (log *K*_ow_ = 4.72) and 1-dodecanol (log *K*_ow_ = 5.13) were investigated. It is clearly seen (Fig. 3(a)) that the most hydrophobic solvent, 1-dodecanol, was the most efficient to extract the Co(ii)–PV complex as it gave the highest absorbance. In addition, it was observed that 1-dodecanol provided good phase separation compared to the others. Consequently, 1-dodecanol was chosen for further studies.

To optimize the volume of the extraction solvent, different volumes of 1-dodecanol from 200 μL to 450 μL were investigated. Fig. 3(b) depicts the effect of extraction solvent on extraction efficiency (as absorbance), volumes less than 300 μL was insufficient to extract all of the Co(ii)–PV complex, while the volumes larger than 400 μL showed the dilution effect. The volume of 300 μL that produced the highest absorbance was chosen for the subsequent experiments.

#### Effect of type and volume of the dispersive solvent

**Fig. 4:**
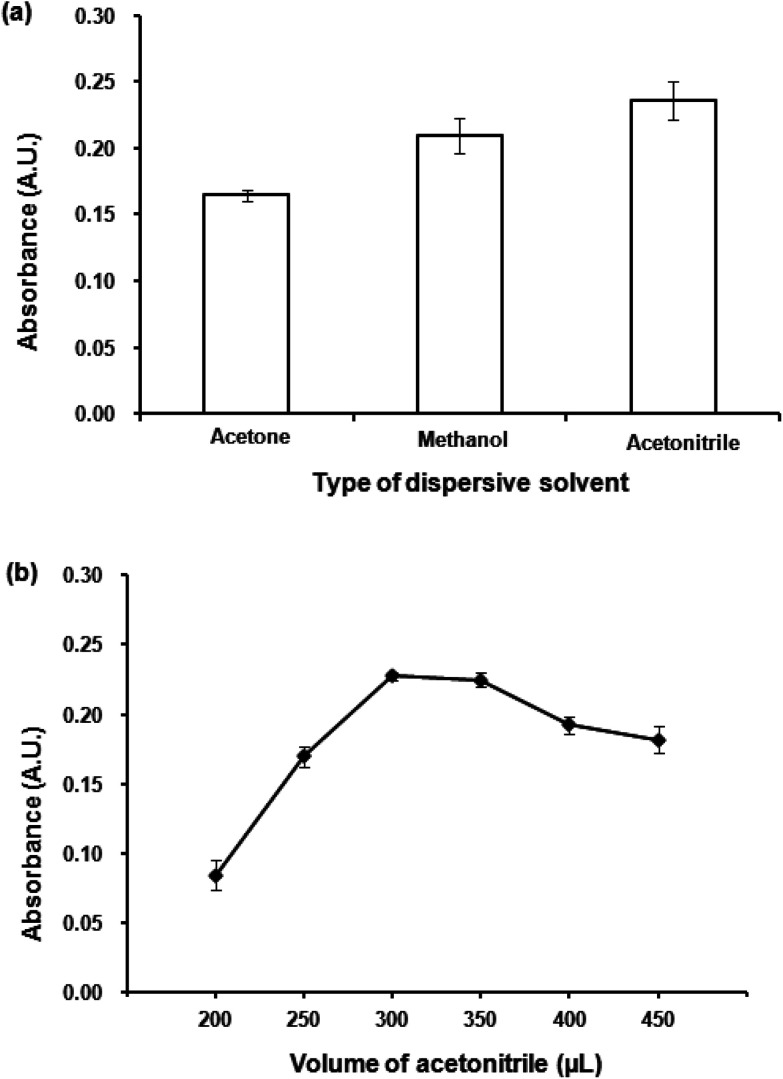
(a) Effect of dispersive solvent. Conditions: 300 μL 1-dodecanol as extraction solvent, 300 μL dispersive solvent, 0.02 mmol L^−1^ CTAB, vortex time at 3200 rpm for 40 s. (b) Effect of dispersive solvent volume. Conditions: as described in (a) except volumes of acetonitrile were varied.

The role of a dispersive solvent is to facilitate the dispersion of an extraction solvent into tiny droplets in an aqueous solution, resulting in a large contact area between the extraction solvent and aqueous solution, enabling an easy transfer of analytes into the extraction solvent droplets. A good dispersive solvent should be miscible in both the extraction solvent and aqueous solution.^40,41^ Three dispersive solvents including acetone, methanol and acetonitrile were investigated. The results (Fig. 4(a)) show that the highest absorbance (0.24 AU) was obtained with the use of acetonitrile as a dispersive solvent, compared with the extraction without dispersive solvent (0.14 AU).

Different volumes acetonitrile (200–450 μL) were studied. Fig. 4(b) shows the dependence of absorbance upon the volume of acetonitrile. Low volume of acetonitrile provided insufficient contact area between the extraction solvent and aqueous solution preventing a good formation of the cloudy solution. The highest absorbance was obtained at 300 μL, after that an increase in acetonitrile volume resulted in a decrease in absorbance. Large volumes of the dispersive solvent increase the solubility of the analytes in the aqueous phase, leading to a decrease in extraction efficiency resulted from a reduction in distribution coefficient.^42^ Therefore, 300 μL of acetonitrile was selected as the optimum volume.

#### Effect of type and amount of salt

**Fig. 5:**
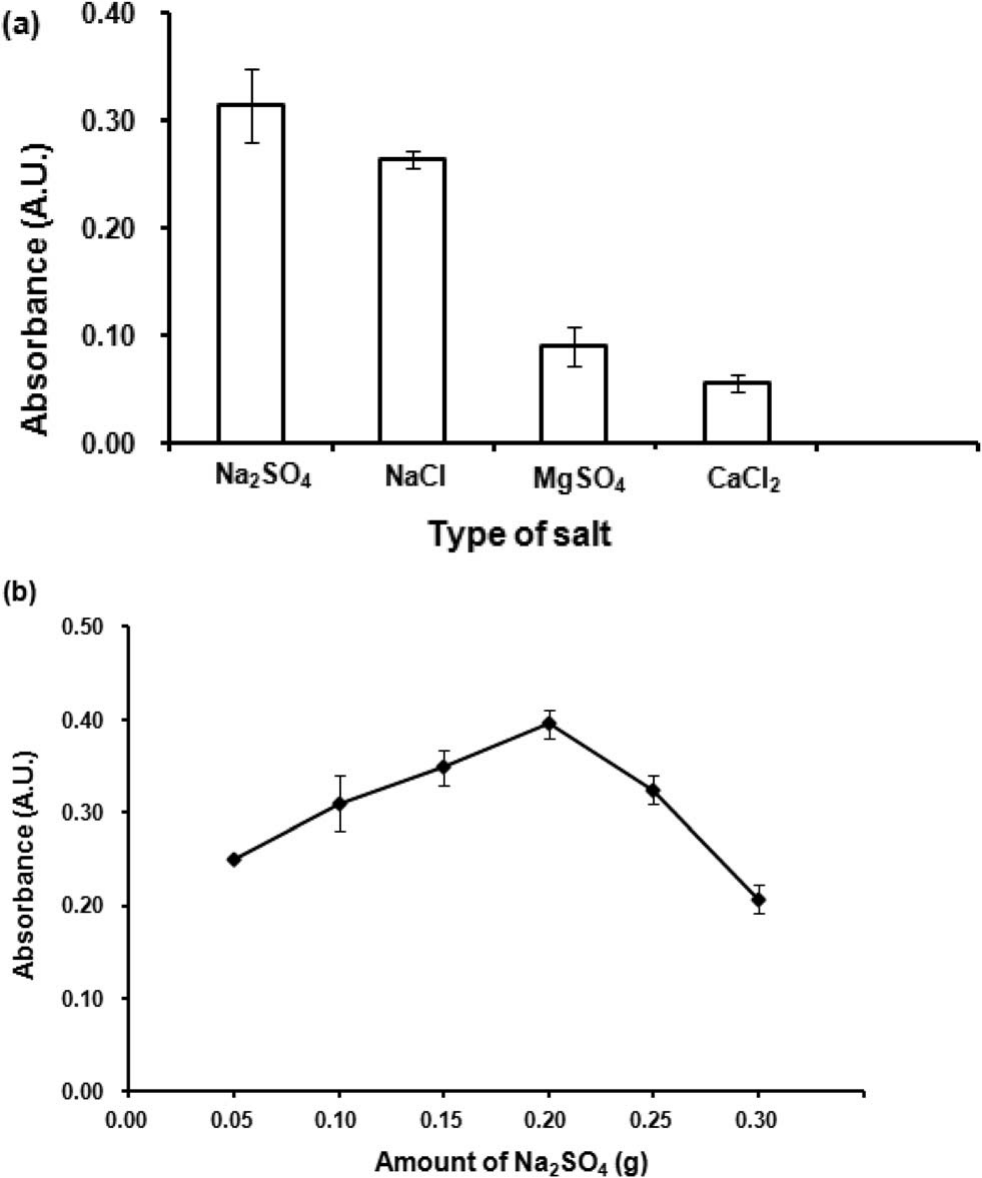
(a) Effect of salt addition. Conditions: 300 μL 1-dodecanol as extraction solvent, 300 μL acetonitrile as dispersive solvent, 0.02 mmol L^−1^ CTAB, 0.1 g (1.0% w/v) salt, vortex at 3200 rpm for 40 s. (b) Effect of amount of salt. Conditions: as described in (a) except amounts of Na_2_SO_4_ were varied.

Due to salting-out effect, the addition of salt decreases the solubility of the analytes in an aqueous solution, resulting in an improvement of their transfer into an organic phase.^18,43,44^ In addition, salt addition decreases the solubility of the extraction phase in the aqueous solution, which facilitates phase separation.^16^ Thus, after the extraction step, phase separation is easily obtained sidestepping centrifugation. In this study, various salts with a weight of 0.1 g (1.0% (w/v)) including sodium chloride (NaCl), sodium sulfate (Na_2_SO_4_), magnesium sulfate (MgSO_4_), calcium chloride (CaCl_2_) were studied. It is clearly seen from Fig. 5(a) that Na_2_SO_4_ gave the highest absorbance.

Effect of Na_2_SO_4_ amount was then studied by varying amounts of Na_2_SO_4_ from 0.05 to 0.3 g. The results (Fig. 5(b)) indicated that 0.2 g Na_2_SO_4_ gave the highest absorbance. Beyond this point, it was found that higher amounts of Na_2_SO_4_ could not improve the extraction efficiency. A high amount of salt increases viscosity of the aqueous phase, thus obstructing the migration of the analytes into the organic extraction phase. Therefore, 0.2 g Na_2_SO_4_ was selected for further studies.

#### Effect of vortex extraction time

Besides the dispersive solvent, it has been reported that a vortex agitator increases contact area between the extraction solvent and aqueous solution, resulting in an improvement of extraction efficiency.^45,46^ In the present study, the vortex rotational speed was fixed at 3200 rpm and the vortex time was varied. The result indicated that increasing vortex time provided higher absorbance and it demonstrated that the equilibrium was achieved after only 20 s (data not shown). Based on these results, the optimal extraction time expressed as vortex time was 20 s.

### Quantitative analysis and method validation

The analytical performances and validation of the proposed method were then investigated including linear range, limit of detection (LOD), limit of quantification (LOQ), precisions (intra-day and inter-day) and interference study. The LOD and LOQ were calculated as Co(ii) concentration giving a signal equal to 3 SD and 10 SD, respectively, where SD is the standard deviation obtained from the measurement of ten blank samples. As summarized in Table 1, good linearity was observed in the range of 0.1–10.0 mg L^−1^ with *R*^2^ = 0.9995. LOD and LOQ were 0.04 mg L^−1^ and 0.13 mg L^−1^, respectively. High precision was obtained with the RSDs of less than 2.10%. The efficiency of the developed DLLME was evaluated in terms of enrichment factor (EF) as the slope ratio of two calibration curves for analyte with and without the preconcentration procedure (direct analysis). The proposed method provided high EF of 13.5.

**Table 1:** Analytical performance for the determination of Co(ii)–PV complex obtained from without (direct analysis) and with VALS-DLLME analysis

Co(ii)–PV complex	Linearity (mg L^−1^)	Linear equation	*R* ^2^	LOD (mg L^−1^)	LOQ (mg L^−1^)	EF	Intra-day (*n* = 6), %RSD	Inter-day (*n* = 6 × 6 days), %RSD
Direct analysis	1.0–10.0	*Y* = 0.0101*X* + 0.0003	0.9996	0.45	1.50	—	1.86	1.98
With VALS-DLLME	0.1–10.0	*Y* = 0.1350*X* + 0.1459	0.9995	0.04	0.13	13.5	2.10	2.35

The selectivity of the proposed spectrophotometric method was determined by adding different amounts of potential interfering species into 1 mg L^−1^ standard Co(ii) before the analysis. The tolerance limit was taken the concentration of the interfering species giving an error of absorbance lower than ±5%. The obtained results are summarized in Table 2. It can be classified into two groups *i.e.* the ions which increased the absorbance (positive bias) including Mg(ii), Mn(ii), Ni(ii) and Fe(ii) and the negative bias (decreased the absorbance) such as Cu(ii), Cd(ii) and Zn(ii), Sn(ii), Al(iii), Fe(iii) and Sb(iii). The studied ions at various concentrations affected the detection of Co(ii), notifying that the tolerance limits for studied metal ions including Ni(ii), Fe(ii), Cu(ii), Cd(ii) and Zn(ii) were higher than 10 mg L^−1^, while those for Mg(ii), Mn(ii), Sn(ii), Al(iii), Fe(iii) and Sb(iii) were higher than 30 mg L^−1^.

**Table 2:** Effect of foreign metal ions on the determination of Co(ii) by the proposed method

Foreign metal ion	Tolerance limit (mg L^−1^)
Cu(ii)	10.1
Fe(ii)	10.2
Zn(ii)	11.1
Ni(ii)	12.4
Cd(ii)	13.2
Mg(ii)	31.0
Mn(ii)	31.2
Al(iii)	32.0
Fe(iii)	34.1
Sn(ii)	35.2
Sb(iii)	35.4

### Application to real samples

The proposed method was applied to determine Co(ii) residue in water samples (tap water, ground water, surface water, agricultural water and wastewater), green leaf vegetables (Chinese cabbage, mint, spinach, cabbage and kale) and five vitamin B_12_ tablets (sample no. I, II, III, IV and V). Co(ii) was not detected in any water or green leaf vegetable samples. However, Co(ii) was detected in some vitamin B_12_ in the range of 0.036–0.729 mg g^−1^. These results were in accordance with those obtained from FAAS (Tables 3–5).

**Table 3:** The determination and recovery of Co(ii) in water samples by the proposed method and FAAS (*n* = 3)

Sample	Spiked (mg L^−1^)	VALS-DLLME			FAAS		
		Found (mg L^−1^)	Recovery (%)	RSD (%)	Found (mg L^−1^)	Recovery (%)	RSD (%)
Tap water	0.0	ND^a^	—	—	ND	—	—
	0.5	0.497	99.4	0.50	0.492	98.5	1.23
	1.0	0.978	97.8	2.82	0.987	98.7	1.28
	3.0	2.912	97.1	2.77	3.041	101.4	1.97
Ground water	0.0	ND	—	—	ND	—	—
	0.5	0.483	96.6	0.78	0.489	97.8	2.78
	1.0	0.963	96.3	1.28	0.991	99.1	3.20
	3.0	3.098	103.3	3.18	3.016	100.5	1.21
Surface water	0.0	ND	—	—	ND	—	—
	0.5	0.472	94.4	1.40	0.479	95.8	1.90
	1.0	0.973	97.3	3.41	0.982	98.2	2.07
	3.0	2.892	96.4	1.18	3.104	103.5	3.42
Agricultural water	0.0	ND	—	—	ND	—	—
	0.5	0.481	96.2	2.63	0.487	97.3	1.63
	1.0	0.982	98.2	1.42	0.987	98.7	2.57
	3.0	2.875	95.8	2.79	3.006	100.2	0.71
Wastewater	0.0	ND	—	—	ND	—	—
	0.5	0.462	92.4	1.63	0.467	93.5	0.51
	1.0	0.971	97.1	3.20	0.985	98.5	3.30
	3.0	2.975	99.2	3.78	3.011	100.4	3.78

a ND: not detected.

**Table 4:** The determination and recovery of Co(ii) in green leaf vegetable samples by the proposed method and FAAS (*n* = 3)

Sample	Spiked (mg g^−1^)	VALS-DLLME			FAAS		
		Found (mg g^−1^)	Recovery (%)	RSD (%)	Found (mg g^−1^)	Recovery (%)	RSD (%)
Mint	0.00	ND^a^	—	—	ND	—	—
	0.25	0.246	98.4	0.78	0.249	99.7	1.21
	0.50	0.495	99.0	1.67	0.496	99.2	2.65
	1.50	1.507	100.5	1.87	1.512	100.8	2.13
Spinach	0.00	ND	—	—	ND	—	—
	0.25	0.226	90.2	0.98	0.228	91.3	0.59
	0.50	0.491	98.2	2.13	0.507	101.3	1.24
	1.50	1.503	100.2	2.43	1.521	101.4	2.57
Kale	0.00	ND	—	—	ND	—	—
	0.25	0.236	94.2	2.53	0.248	99.1	0.76
	0.50	0.489	97.9	2.36	0.491	98.1	1.43
	1.50	1.554	103.6	1.89	1.523	101.5	0.98
Cabbage	0.00	ND	—	—	ND	—	—
	0.25	0.242	96.8	1.09	0.246	98.4	1.32
	0.50	0.496	99.1	2.34	0.506	101.2	2.36
	1.50	1.456	97.1	1.54	1.556	103.7	2.41
Chinese cabbage	0.00	ND	—	—	ND	—	—
	0.25	0.241	96.4	1.24	0.244	97.6	0.97
	0.50	0.493	98.6	2.32	0.503	100.6	1.23
	1.50	1.498	99.9	1.14	1.515	101.1	1.09

a ND: not detected.

**Table 5:** The determination and recovery of Co(ii) in vitamin B_12_ samples by the proposed method and FAAS (*n* = 3)

Sample	Spiked (mg g^−1^)	VALS-DLLME			FAAS		
		Found (mg g^−1^)	Recovery (%)	RSD (%)	Found (mg g^−1^)	Recovery (%)	RSD (%)
No. I (1 tablet = 0.1509 g)	0.000	0.729	—	—	0.762	—	—
	0.165	0.868	94.1	1.24	0.888	94.0	2.49
	0.331	1.054	99.6	2.43	1.067	101.8	1.57
	0.994	1.736	101.1	1.09	1.749	102.3	0.98
No. II (1 tablet = 0.1374 g)	0.000	0.699	—	—	0.706	—	—
	0.182	0.859	86.0	1.54	0.888	99.0	1.26
	0.364	1.077	102.7	0.98	1.084	103.0	2.37
	1.092	1.776	99.7	2.32	1.849	104.3	1.57
No. III (1 tablet = 0.1270 g)	0.000	0.701	—	—	0.702	—	—
	0.197	0.882	92.0	1.43	0.898	98.0	1.43
	0.394	1.087	99.5	1.57	1.118	104.5	1.89
	1.181	1.882	100.7	0.69	1.953	105.7	0.94
No. IV (1 tablet = 0.3056 g)	0.000	0.111	—	—	0.115	—	—
	0.081	0.187	92.0	2.12	0.193	93.1	2.31
	0.164	0.272	97.0	3.45	0.275	99.0	1.54
	0.491	0.605	100.7	1.34	0.612	101.0	0.92
No. V (1 tablet = 0.3924 g)	0.000	0.036	—	—	0.038	—	—
	0.064	0.097	96.0	1.57	0.092	84.0	1.27
	0.127	0.163	98.5	2.58	0.168	102.4	0.93
	0.382	0.415	99.3	2.63	0.426	101.3	1.38

The accuracy of the proposed method was also investigated as recovery by spiking known concentrations at three levels of Co(ii) into sample solutions before acid digestion and ashing for vitamin B_12_ and vegetable sample, respectively. The obtained solutions were then subjected to VALS-DLLME and spectrophotometric analysis. All experiments were performed in triplicate. The recoveries of water, vegetable and vitamin B_12_ samples (Tables 3–5) were obtained in the range of 92.4–103.3%, 90.2–103.6% and 86.0–102.7% with RSD less than 3.78, 2.53 and 3.45%, respectively. In addition, the accuracy of the proposed method was studied by comparing the results with those obtained from FAAS. The results indicated an insignificant difference (*p* = 0.05) between the proposed method and FAAS.

## Conclusions

A simple and sensitive spectrophotometric method has been successfully developed for the determination of Co(ii) in water, green leaf vegetable and vitamin B_12_ samples. The method is based on the complexation of Co(ii) with pyrocatechol violet (PV) and the preconcentration of Co(ii)–PV complex by vortex-assisted low density solvent and surfactant based dispersive liquid–liquid microextraction (VALS-DLLME) before measurement of the absorbance at visible wavelength. The proposed method provided high precision, low LOD and high accuracy. Moreover, the method employs visible spectrophotometer, an unsophisticated instrument, providing an economical alternative to FAAS for the determination of Co(ii) in real samples.

## Conflicts of interest

There are no conflicts to declare.

## Supplementary Material
